# Brain Metastases Management in Oncogene-Addicted Non-Small Cell Lung Cancer in the Targeted Therapies Era

**DOI:** 10.3390/ijms23126477

**Published:** 2022-06-09

**Authors:** Elisa De Carlo, Elisa Bertoli, Alessandro Del Conte, Brigida Stanzione, Eleonora Berto, Alberto Revelant, Michele Spina, Alessandra Bearz

**Affiliations:** 1 Dipartimento di Oncologia Medica, Centro di Riferimento Oncologico di Aviano (CRO) IRCCS, 33081 Aviano, Italy; elisa.decarlo@cro.it (E.D.C.); elisa.bertoli@cro.it (E.B.); alessandro.delconte@cro.it (A.D.C.); brigida.stanzione@cro.it (B.S.); eberto@cro.it (E.B.); mspina@cro.it (M.S.); 2 Department of Medicine (DAME), University of Udine, 33100 Udine, Italy; 3 Dipartimento di Radioterapia, Centro di Riferimento Oncologico di Aviano (CRO) IRCCS, 33081 Aviano, Italy; alberto.revelant@cro.it

**Keywords:** NSCLC, oncogenic biomarkers, brain metastases, targeted therapies

## Abstract

The therapeutic landscape in patients with advanced non-small-cell lung cancer harboring oncogenic biomarkers has radically changed with the development of targeted therapies. Although lung cancers are known to frequently metastasize to the brain, oncogene-driven non-small-cell lung cancer patients show a higher incidence of both brain metastases at baseline and a further risk of central nervous system progression/relapse. Recently, a new generation of targeted agents, highly active in the central nervous system, has improved the control of intracranial disease. The intracranial activity of these drugs poses a crucial issue in determining the optimal management sequence in oncogene-addicted non-small-cell lung cancer patients with brain metastases, with a potential change of paradigm from primary brain irradiation to central nervous system penetrating targeted inhibitors.

## 1. Introduction

Lung cancer is one of the most commonly diagnosed cancers (11.6% of all new tumors) [1]. Despite a decline in the death rate in recent years, lung cancer is still the leading cause of cancer deaths [2]. Non-small-cell lung cancer (NSCLC) accounts for 85% of lung cancers, with 60–70% of patients presenting at either stage III B or stage IV of the disease [3].

Brain metastases (BMs) are a common complication in a wide range of cancers, but they are particularly common among NSCLC patients. Indeed, the incidence rate of BMs at diagnosis is 10–20% and up to 40% during the course of the disease [4,5,6].

In recent times, the lifetime incidence of the central nervous system (CNS) metastases in NSCLC has increased as a result of both improved neuro-imaging techniques by the use of magnetic resonance imaging and furthermore of an increase in patient survival because of better systemic control of extracranial disease.

In patients with metastatic lung cancer, BMs are associated with inferior health-related quality of life (QoL) and poor prognosis (life expectancy ranging between 3 and 13 months) [7].

Historically, CNS has been considered a pharmacological sanctuary because of the physical and chemical characteristics of the blood–brain barrier (BBB), a diffusion barrier essential for CNS function. The continuous tight junctions that join the endothelial cells in the brain capillaries limit the influx of circulating factors from blood to brain [8].

Local treatments have traditionally been the cornerstone of BMs management. Surgical resection (SR), stereotactic radiosurgery (SRS), and whole-brain radiation therapy (WBRT) have been the primary treatment modalities.

Brain irradiation can significantly impair BBB integrity.

**Figure 1:**
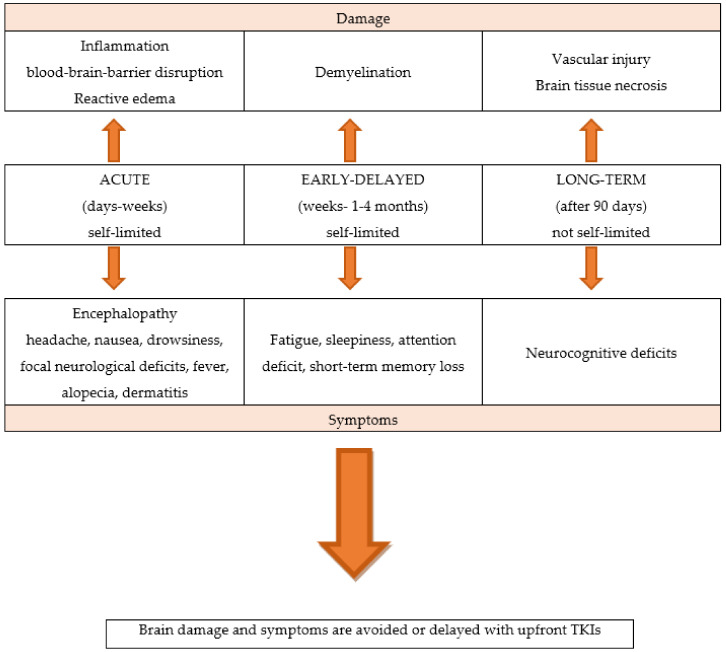
The neurological toxicities of brain radiotherapy.

WBRT is associated with a significant rate of neurological toxicities, and acute, early-delayed, and late effects. Unlike acute neurological symptoms, which are usually reversible, long-term effects appear several months or years later and are generally irreversible [9,10,11]. Partial brain RT may also have late effects on cognitive function, although the risk is lower than with WBRT. Figure 1

The selection of local treatment is based on the number of BMs, the size or location of BMs, the symptoms of encephalic disease, and the status of extracranial metastases.

For patients with symptomatic or a limited number of BMs, who have a controlled primary disease or are suitable for radical treatment, local therapy with a neurosurgical resection or with SRS is recommended. Instead, WBRT is considered for pluri-metastatic CNS disease [12].

SR is often the standard of care (SoC) for solitary or symptomatic brain metastasis, and it can provide immediate and effective relief from symptomatic mass effects [13,14]. The combination of the neurosurgical resection of solitary brain metastasis and postoperative RT favored the combination treatment. A phase III randomized control trial compared post-operative SRS to the surgical cavity with WBRT in patients who underwent resection of a single brain metastasis and demonstrated a lower probability of deterioration in cognitive function and no difference in overall survival (OS) [15]; thus, adjuvant SRS to the surgical resection bed should be considered the preferred local therapy.

A systematic review did not show a significant outcome difference between SR and SRS in patients with single brain metastasis [16].

SRS alone has become the SoC for patients with a good performance status, who cannot undergo resection and/or have a limited number of BMs (1–4 BMs) [17].

SRS allows many precisely focused radiation beams, improving healthy tissue preservation and less cognitive decline.

In past decades, WBRT was the most widely used local treatment for the management of patients with multiple BMs. The radiation schedules include a classical dose of 30 gray (Gy) in 10 fractions or a short course of 20 Gy in 5 fractions, showing similar efficacy [18].

In the last few years, the role of WBRT is declining because of the potential cognitive deficits and the limited clinical benefit compared with best supportive care [19,20].

Therefore, because of its high risk of cognitive function deterioration and unproven survival benefit, the WBRT could be an upfront treatment option for patients with symptomatic BMs; alternatively, a wait-and-see approach could be adopted.

Cytotoxic chemotherapy (CT) plays a limited role in controlling BMs because of the drugs’ inability to cross the BBB and penetrate the CNS [21].

Platinum*-*based regimens have been the most commonly used therapy for metastatic NSCLC. These CT regimens demonstrated low systemic response rate (RR) in a few brain RT-naïve patients [22].

Thus, upfront CT could represent the better option then WBRT in NSCLC patients with multiple asymptomatic BMs, who are not eligible for SRS. Therefore, WBRT could be reserved for symptomatic patients with good performance status or for intracranial non-responders.

In recent years, both single-agent immune checkpoint inhibitors (ICIs) and a combination of CT plus ICIs have shown better efficacy than platinum*-*based regimens in NSCLC patients, who do not harbor a driver oncogene alteration in randomized phase III clinical trials. A future critical challenge is knowing how to identify NSCLC patients with CNS disease who benefit from ICIs and the potential of combining radiation with ICIs.

**Figure 2:**
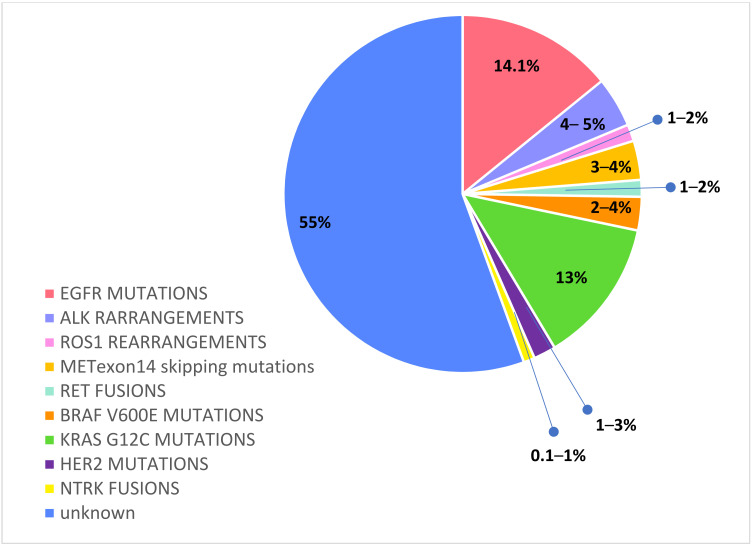
Druggable molecular alterations with tailored treatments in oncogene-addicted NSCLC.

Recently, routine *molecular testing* has become the SoC for determining the optimal treatment of newly diagnosed advanced or metastatic NSCLC patients. In particular, a range of predictive and prognostic biomarkers have been identified in adenocarcinoma: epidermal growth factor receptor (EGFR) mutations, anaplastic lymphoma kinase (ALK) rearrangements, c-ros oncogene 1 (ROS1) rearrangements, v-Raf murine sarcoma viral oncogene homolog B1 (BRAF) mutations, Kirsten rat sarcoma viral oncogene homologue (KRAS) mutations, neurotrophic receptor tyrosine kinase (NTRK) 1/2/3 rearrangements, rearranged during transfection (RET) rearrangements, N-methyl-N′-nitroso-guanidine human osteosarcoma transforming gene (MET) exon14 skipping mutations, and activating human epidermal growth factor receptor 2 (HER2) mutations [23,24,25]. Figure 2

According to previous reports, driver oncogenic alterations, such as EGFR, ALK, ROS1, RET, NTRK, and HER2, have a higher frequency in never smokers, younger age, females, and Asian NSCLC patients, with a tendency to early metastasis and to brain dissemination [26].

The sequencing of the human genome has permitted one to characterize different molecular subgroups of lung cancer patients, who are grouped under the definition of oncogene-addicted NSCLC.

The identification of an ever-increasing number of potentially druggable molecular alterations has led to the development of tailored treatments, which are tyrosine kinase inhibitors (TKIs), with remarkable results in terms of intracranial disease control and OS. Multiple randomized phase III studies demonstrated the superiority of first-line targeted therapies over platinum-based CT for oncogene-addicted NSCLC patients.

Compared to non-oncogenic-driven NSCLC, the prognosis of mutated patients has continuously improved. NSCLC patients with oncogenic-driven mutations are more likely to develop BMs because of the better control of extracranial disease and prolonged survival.

Given the significant CNS activity of novel targeted agents, there is growing evidence that upfront treatment with tailored systemic therapies can sufficiently control BMs.

There are several reasons why local brain irradiation can be deferred in favor of first-line next-generation TKIs:

- The newer generation targeted systemic therapies have demonstrated far greater CNS penetration than CT or older targeted agents; the newer molecular targeted agents are liposoluble compounds with low molecular weight and can cross the BBB; furthermore, they have the ability to penetrate cerebrospinal fluid (CSF).
- Oncogene-addicted NSCLC patients are living several years rather than only a few months, allowing for more time for BMs to develop, as well as for adverse effects from prior RT to manifest.
- These factors lead to a treatment strategy shift, privileging brain penetrant TKIs systemic therapies over local treatments, maintaining patient QoL by minimizing the RT-related consequences.

In the era of targeted therapies, the management of BMs is a challenging issue. It remains unclear whether it is reasonable enough to defer RT until the intracranial progression is noted in patients on TKIs; therefore, the question of how and when to perform brain RT remains open.

Studies on the clinical efficacy of RT combined with TKIs for patients with CNS metastases are very limited and largely retrospective; most adopt only WBRT, and many prospective trials recruit relatively small numbers of patients without considering oncogenic mutational status [27,28,29]. Furthermore, data for patients with molecular driver alterations other than those EGFR are even more scarce, and the efficacy of combined RT with these agents is only anecdotal [30].

Perspective trials appropriately designed to assess the optimal timing of brain RT are necessary.

In this review, we report the literature data on the intracranial activity of targeted agents for specific oncogenic driver biomarkers. We also report the evolving management approach, suggesting upfront targeted therapies versus brain RT in NSCLC patients with driver mutations and CNS metastases at diagnosis.

## 2. Egfr Mutations

Activated EGFR mutations, predominantly exon 19 deletions and exon 21 L858R mutations, occur in approximately 14.1% of Caucasian NSCLC [31].

Among EGFR mutated NSCLC patients, BMs have an increased frequency, considering baseline incidence ranging from 23% to 32% [32,33,34,35] and a further risk of intracranial progression of about 15–20% during first-generation TKIs treatment [36,37].

These data reflect a pharmacokinetic failure of the first- and second- generation EGFR TKIs to penetrate the brain. Though erlotinib, gefitinib, and afatinib have intracranial activity, these agents have a limited BBB penetration, and they are detected in CFS only at a low concentration, in the 1 to 5 percent range of what is observed in the serum [38,39,40,41].

By contrast, third-generation irreversible TKI osimertinib achieves a greater intracerebral concentration and has shown high intracranial response rates, even against leptomeningeal carcinomatosis [42,43,44].

Osimertinib was first approved in the second-line setting in patients that developed a T790M mutation after failure of a first-generation TKI.

Pooled data from two phase II trials—AURA extension and AURA2—in 50 T790M-positive advanced NSCLC patients with BMs progressed to prior EGFR TKI have demonstrated the significant intracranial activity of osimertinib; CNS objective response rate (ORR) and disease control rate (DCR) were 54% and 92%, respectively, and CNS response was observed regardless of prior brain irradiation [45].

In the randomized phase III AURA 3 trial osimertinib demonstrated significantly greater progression-free survival (PFS) than platinum-based doublet-CT in patients with EGFR T790M advanced NSCLC and progression on prior EGFR-TKI treatment. Among 116 patients with BMs (measurable or not), PFS was longer with osimertinib compared to CT (11.7 vs. 5.6 months, HR 0.32; and 95% CI: 0.15–0.69) and cumulative incidence of CNS progression at 6 months was lower with osimertinib compared to CT (11.5% vs. 28.2%) [46].

Subsequently, osimertinib was approved in the first-line setting on the basis of the randomized phase III FLAURA trial, which evaluated the efficacy of upfront osimertinib versus a SoC EGFR TKI (erlotinib or gefitinib) in treatment-naïve EGFR mutant (exon 19 del or L858R) advanced NSCLC patients [47].

The CNS activity of osimertinib was confirmed in a subset analysis of the randomized phase III FLAURA trial, which evaluated the efficacy of upfront osimertinib versus a SoC EGFR TKI (erlotinib or gefitinib) in treatment-naïve EGFR mutant (exon 19 del or L858R) advanced NSCLC patients. In the preplanned, exploratory analysis (CNS analysis set, *N*  =  128), osimertinib reported that improved CNS RR (66% vs. 43%) and median CNS PFS among patients with measurable and/or non-measurable CNS lesions was longer (not reached vs. 13.9 months, HR 0.48, 95% CI: 0.26–0.86, and *p*  =  0.04). Furthermore, osimertinib reduced the risk of CNS progression in the overall study population (6% vs. 15%), regardless of the presence or absence of known or treated CNS metastases at baseline. Among patients with BMs evaluable for response (*N*  =  41), osimertinib improved the CNS RR (91% vs. 68%) [48].

Data from this analysis show that osimertinib reveal encouraging activity against CNS involvement, with a greater intracranial response and clinical benefit both in preventing or delaying BMs.

Randomized trials comparing upfront osimertinib with brain irradiation are lacking. Although retrospective data indicate that the deferral of RT may be associated with worse outcomes compared with early RT [49,50,51,52,53], those studies were all conducted with earlier-generation EGFR TKIs, which have less intracranial activity than osimertinib.

In sum, the available data suggest that osimertinib demonstrates the greatest CNS activity and prevention of CNS progression, making it the preferred initial treatment option for EGFR-mutated NSCLC with BMs, deferring brain RT and its neurocognitive defects in case of intracranial progression.

## 3. Alk Rearrangements

Patients with ALK gene rearrangements represent approximately about 4–5% of all NSCLC patients [54].

ALK-positive NSCLC patients carry a high risk of developing CNS metastases, as observed in at least 20% of cases at diagnosis [33].

As the treatment of ALK TKIs improves outcomes over CT, the management of CNS metastases has become a relevant therapeutic issue.

The CNS appeared to be a common first site of progression among treatment-naïve ALK patients treated with first-generation TKI crizotinib, the first approved ALK TKI [55].

A retrospective analysis of the crizotinib PROFILE 1005 and PROFILE 1007 trials in patients treated with crizotinib revealed an intracranial RR of 18% among those with untreated BMs and 33% among those with treated BMs. The median intracranial PFS was 5.9 months and 6 months among those with untreated and treated BMs, respectively.

The pharmacokinetic failure is mainly due to its poor BBB penetration [56].

In the case of CNS progression during crizotinib treatment, brain RT and the continuation of crizotinib is the accepted strategy to control BMs and extend the survival benefit [55].

Preclinical studies have demonstrated that combining RT and ALK-TKIs may affect tumor growth and microvascular density; however, data of this synergistic strategy are scarce [57].

Compared to crizotinib, the newer generation ALK TKIs have been developed to enhance CNS exposure, crossing BBB and achieving a higher concentration in CFS.

In front-line setting second-generation ALK inhibitors, alectinib, brigatinib, ceritinib, and third-generation ALK inhibitor lorlatinib have demonstrated promising intracranial activity.

Alectinib demonstrated an extremely high penetration rate across the BBB, which might be explained also by the fact that alectinib, unlike crizotinib, is not a substrate for P-glycoprotein [58].

Alectinib was the first TKI approved in the crizotinib-resistance setting.

A pooled analysis of two phase II trials, including 136 patients with BMs (70% had prior RT), have demonstrated great intracranial activity of alectinib, with CNS RR of 64% in patients with measurable disease [59]. Further support for the intracranial efficacy of alectinib comes from the phase III ALUR trial, in which crizotinib and CT-resistant patients were randomly assigned to alectinib or single-agent CT. Among 24 patients with measurable BMs, alectinib demonstrated greater intracranial ORR over CT (54% vs. 0%) [60].

In first-line setting, the CNS activity of alectinib over crizotinib comes from two phase III randomized trials, ALEX and J-ALEX trial.

In ALEX trial, among patients with measurable BMs, alectinib demonstrated greater PFS (27.7 months and 7.4 months), intracranial RR (81% vs. 50%), and CNS median duration of response (mDoR) (17.3 and 5.5 months). Patients with previously irradiated BMs had higher intracranial RR (86% vs. 79%) than patients without previous RT [61,62].

The time to CNS progression was significantly longer with alectinib (HR 0.16; 95% confidence interval [95% CI] 0.1–0.28; *p* < 0.001). The 12-month cumulative incidence rate of CNS progression in the alectinib and crizotinib groups was 9.4% vs. 41.4%, respectively. Similar results were observed in the Japanese population of the J-ALEX trial [63].

Other next-generation ALK inhibitors also demonstrated activity in the CNS.

Brigatinib has demonstrated systemic and intracranial efficacy over crizotinib in the phase III ALTA-1L trial in the front-line setting [64]. Among 275 patients, 90 had BMs at baseline. The confirmed CNS RR among patients with measurable BMs (39) was 78% with brigatinib (95% CI, 52 to 94) vs. 29% with crizotinib (95% CI, 11 to 52). The estimated rate of 12-month survival without intracranial progression among patients with baseline BMs in brigatinib and crizotinib arms was 67% and 21%, respectively.

Ceritinib has been compared with CT but not crizotinib in a first-line setting.

In the preliminary results of the ASCEND-7 trial, which evaluated ceritinib in patients with newly diagnosed BMs, among 44 patients with no prior brain RT or ALK TKI, the intracranial RR was 51.5% (33.5–69.2%) and the median duration of intracranial response was 7.5 months [65].

The third generation Lorlatinib is a highly potent ALK TKI with excellent BBB penetration. Lorlatinib received approval for the second- or third-line treatment of ALK-positive metastatic NSCLC (after alectinb or ceritinib failure) on the basis of the results of a phase I trial in a pretreated ALK-positive and ROS1-rearranged patient population. In this phase I trial, RR reported with lorlatinib in patients with measurable and non-measurable BMs reached 39% and 31%, respectively [66].

In the phase II trial, lorlatinib confirmed the CNS activity in patients with measurable BMs, with an intracranial RR of 66.7% in treatment-naïve patients, 87% in crizotinib-refractory patients, 63% in patients treated with at least one previous ALK TKI, and 53.1% in those who received two or more ALK inhibitors [67].

In the first-line setting, the CNS activity of lorlatinib over crizotinib comes from the phase III CROWN trial compared head-to-head lorlatinib with crizotinib in untreated ALK-positive NSCLC patients. Among 30 patients with BMs, 82% of patients (14/17) treated with lorlatinib versus 23% of patients (3/13) who received crizotinib achieved an intracranial response; 71% of those treated with lorlatinib had an intracranial complete response [68].

For patients who experienced isolated CNS progression in the course of 2° generation ALK-TKI, with stable disease extracranially, switching to a more potent ALK inhibitor is an alternative approach to brain RT. 

## 4. Ros1 Rearrangements

The rearrangements of the ROS1 gene has been identified in approximately 1–2% of NSCLC patients [69]. BMs are common in treatment-naive stage IV ROS1-positive NSCLC [70,71].

Crizotinib was the first-in-class TKI for metastatic ROS1 fusion-positive NSCLC [72,73]. BMs are a common first site of progression in ROS1-positive patients who are taking crizotinib because this agent has poor CNS penetration and is a substrate of P-glycoprotein, a membrane protein that pumps xenobiotics out of CNS [56].

Newer TKIs, including entrectinib, repotrectinib, and lorlatinib, have been developed to penetrate the BBB.

Entrectinib is approved for NSCLC patients harboring ROS1 alterations and is currently the preferred agent in those with BMs.

Entrectinib is a low-molecular-weight and potent ROS1 inhibitor, specifically designed to cross BBB. Preclinical models have confirmed that entrectinib is a weak substrate of P-glycoprotein, achieving high concentrations in CFS [74,75].

Entrectinib has demonstrated CNS activity in crizotinib-naïve, ROS1-positive patients [76]. A pooled analysis of three trials, one phase I trial and two phase II trials (ALKA-372-001, STARTRK-1, and STARTRK2), evaluated the activity of entrectinib in 53 ROS1 TKI-naive patients. Among 24 patients with measurable baseline BMs, the intracranial ORR was 79% (n = 19; IC 95%: 57.9–92.9), the median intracranial PFS was 12.0 months (IC 95%: 6.2–19.3), and the median intracranial DoR was 12.9 months [77].

The encouraging systemic and intracranial activity in patients with NSCLC harboring a ROS1 rearrangement led to the approval of entrectinib. Based on its excellent overall efficacy and superior activity within the CNS, it is the preferred upfront option for patients with ROS1-translocated NSCLC.

For patients who have relapsed on front-line crizotinib, the third-generation TKI lorlatinib is an option (although lorlatinib is off-label in this setting). The available data support great intracranial activity with lorlatinib. In a phase I study evaluating lorlatinib, five patients had measurable BMs; three of five patients (60%) had intracranial objective responses, two of whom had experienced disease progression on prior crizotinib [66]. Similarly, in the phase II study of lorlatinib, the intracranial ORR for TKI-naive and crizotinib-resistant patients was 64% and 50%, respectively [78].

The investigational agent repotrectinib demonstrated promising efficacy for patients with ROS1-positive NSCLC in a subgroup analysis of TRIDENT-1 trail. This study included seven patients with measurable BMs at baseline. Among three TKI-naïve patients the intracranial ORR was 100%, and among four TKI-pretreated patients, the intracranial ORR was 50% [79]. In both groups, the mDoR was 5.5 months. Table 1.

## 5. Met Exon-14-Skipping Mutations

METexon14 skipping mutations (METex14) represent 3–4% of NSCLC [80].

Capmatinib is a highly selective and potent MET inhibitor that crosses the BBB, approved by the FDA for the treatment of patients with METex14-positive advanced NSCLC based on the multi-cohort phase II GEOMETRY mono-1 trial. In preliminary results of this trial, there were 13 patients with evaluable baseline BMs [81]. Upon treatment with capmatinib, 7 of the 13 patients (54%) had an intracranial response, 4 of whom had a complete response.

Tepotinib is another FDA-approved agent for METex14-positive patients. In the VISION trial, this agent demonstrated intracranial activity among 11 patients with BMs (all of whom were nontarget lesions), with an RR of 55% and a mDoR of 9.5 months [82].

## 6. Ret Fusions

RET gene rearrangements are found in 1–2% of NSCLC [83]. Novel-RET directed targeted therapies, either selpercatinib and pralsetinib, are selective RET inhibitors demonstrating great overall and intracranial efficacy.

A pre-planned analysis of the multicohort, open-label, phase I/II LIBRETTO-001 trial has reported the intracranial efficacy of selpercatinib among heavily pretreated RET fusion-positive patients with CNS disease at baseline. Among 22 patients with measurable BMs, intracranial ORR was 82%, including 23% with a complete response; 18% of patients exhibited stable disease as the best response. Because all the patients achieved a tumor response or disease stabilization, the intracranial DCR was 100%. Among the subset of eight patients with measurable BMs and prior brain RT, the intracranial ORR was 75% (6/8); for patients without prior cranial RT, it was 86% (12 of 14 patients responding; 95% CI, 57–98) [84].

Similarly, Pralsetinib has demonstrated significant intracranial activity, with an intracranial RR of 78% (7/9) of patients with baseline measurable BMs in an early-phase clinical study [85].

Furthermore, there have been documented responses to selpercatinib in leptomeningeal disease and in the case of intracranial progression after prior systemic and local therapies [86,87].

## 7. Braf V600E Mutations

Activating mutations in BRAF occur in approximately 2–4% of NSCLC patients. The most common BRAF mutation is V600E, which results in a glutamate substitution for valine at codon 600 [88].

The combination therapy of BRAF inhibitor Dabrafenib and the MEK inhibitor Trametinib is the approved first-line therapy for BRAF V600E-positive NSCLC patients [89,90].

Data regarding intracranial disease control are not available; limited clinical practice data have described the intracranial activity of the combination therapy in NSCLC patients [91]. However, this TKIs association has reported CNS efficacy in BRAF V600E-positive melanoma, making this activity highly probable in NSCLC patients with this molecular driver alteration. Real*-*world evidence is useful to inform clinical practice.

## 8. Kras G12C Mutations

KRASG12C (glycine 12 to cysteine) mutation has been identified in approximately 13% of NSCLC. Recently, the KRAS G12C mutation has been identified as a targetable oncogenic mutation that confers sensitivity to covalent inhibitors [92,93].

Sotorasib is a first-in-class, selective, irreversible targeted agent with regulatory approval by the FDA for KRAS G12C-mutated locally advanced or metastatic NSCLC patients, who have received at least one prior systemic therapy.

In the post-hoc analysis of phase 1/2 CodeBreaK 100 trial, Sotorasib demonstrated a great intracranial response in KRAS G12C-positive NSCLC patients with stable BMs, previously treated with either RT or surgery.

At a median follow-up of 12 months, sotorasib led to an ORR of 25% in patients with baseline BMs compared with 42% in patients without BMs; the DCR was 77.5% vs. 84.1%, respectively; mPFS was 5.3 months (95% CI, 2.7–9.3) vs. 6.7 months (95% CI, 5.3–8.2), respectively [94].

## 9. Her2 Mutations

Mutations in HER2 have been detected in approximately 1% to 3% of NSCLC patients [95]. They usually involve small in-frame insertions in exon 20, but point mutations in exon 20 have also been observed.

In the DESTINY-Lung01 phase II trial, trastuzumab deruxtecan demonstrated durable anticancer activity in previously treated HER2-positive NSCLC patients. CNS surveillance was not performed systematically in all patients; therefore, the data of intracranial activity are not available. However, among the 33 patients with CNS disease, the percentages of patients with a response were similar to those without BMs [96].

## 10. Ntrk Fusions

NTRK gene fusions are very rare and occur at a frequency of ~0.1−1.0% [97]. The oral TRK inhibitors larotrectinib and entrectinib are FDA-treatment-approved and either option is appropriate for advanced or metastatic NTRK fusion-positive NSCLC.

The intracranial efficacy of both targeted agents has been shown in early-phase clinical trials. In small numbers of NTRK fusion-positive NSCLC, entrectinib has shown durable systemic and intracranial efficacy, with responses in 4/6 (67%) patients [98]. A pooled analysis of two clinical trials has also demonstrated the intracranial response to larotrectinib; this small cohort included three NSCLC patients, of which one had a response [99].

## 11. Conclusions

The development of genotype-directed therapies for actionable oncogenic drivers has improved the intracranial and overall response, QoL, PFS, and even OS, for oncogene-addicted NSCLC patients. Therefore, it is crucial to perform broad molecular testing in diagnosis of advanced and metastatic NSCLC in order to define the optimal systemic treatment.

CNS is a common site of metastatic disease in NSCLC harboring driver genetic alterations. In the last few years, the development of CNS-penetrant TKIs has determined a great challenge for the optimal management of oncogenic-driven NSCLC patients with BMs.

The available data lead to a treatment paradigm shift favoring as an initial approach systemic therapy with brain penetrant next-generation inhibitors, which provide excellent control of intracranial disease. The goal is to optimize both OS and QoL, with the high priority of avoiding or deferring brain irradiation and its neurocognitive sequelae.

Based on the available clinical trial data and long OS in patients with asymptomatic CNS disease at diagnosis, the frontline approach with brain-penetrating TKIs alone should be considered with close imaging surveillance for early intervention in non-responding patients. This approach may defer brain RT, avoiding or delaying neurological toxicities associated with irradiation. For patients with symptomatic brain metastases, initial TKI therapy is suggested. Because of the lack of prospective trials comparing frontline TKIs and sequential treatment, upfront RT remains an appropriate alternative.

In cases of brain progression and stable extracranial disease while on TKI therapy, there are two options: switch to another higher CNS penetrating TKI if available or the administration of brain RT followed by the same TKI.

Due to the complexity of oncogene-addicted NSCLC with BMs, a multidisciplinary approach for the best management is required.

The increasing knowledge of lung cancer biology and the development of actionable molecular targets are determining an evolving approach to oncogene-driven NSCLC with CNS disease. However, the optimal timing of TKI treatment and intracranial RT remains to be further confirmed. Real-world data and randomized prospective trials are needed to indicate which patients are most likely to benefit from the combined or sequential use of tailored and radiation therapy.

## Figures and Tables

**Table 1:** The intracranial activity of targeted agents in TKIs-naïve patients.

DRIVER BIOMARKER	BRAIN PENETRANT TKIs	IC ORR
EGFR MUTATIONS	Osimertinib	66%
ALK REARRANGEMENTS	Alectinib Brigatinib Lorlatinib	81% 78% 82%
ROS1 REARRANGEMENTS	Entrectinib Lorlatinib Repotrectinib	79% 64% 100%

Abbreviations: TKIs, tyrosine kinase inhibitors; IC, intracranial; and ORR, intracranial objective response rate.

## Data Availability

Not applicable.
